# Sweet Cherry (*Prunus avium* L.) Response to Self-Regulating Low Energy Clay-Based Irrigation (S.L.E.C.I.) System

**DOI:** 10.3390/plants14223533

**Published:** 2025-11-19

**Authors:** Svetoslav Malchev, Vjekoslav Tanaskovik, Ordan Chukaliev, Daniela Germanova, Georgi Kornov

**Affiliations:** 1 Agricultural Academy, Fruit Growing Institute-Plovdiv, 12 Ostromila Str., 4004 Plovdiv, Bulgaria; dporqzova@yahoo.com (D.G.); joro_kornov@abv.bg (G.K.); 2 Faculty of Agricultural Sciences and Food, University “Ss. Cyril and Methodius”-Skopje, 16th Macedonian Brigade No. 3, 1000 Skopje, North Macedonia; vtanaskovic@fznh.ukim.edu.mk (V.T.); cukaliev@gmail.com (O.C.)

**Keywords:** S.L.E.C.I., irrigation, DIVAGRI project, cherry, clay tubes

## Abstract

In early initial tests, the Self-regulating Low-Energy Clay-based Irrigation (S.L.E.C.I.) has provided convincing results. During the DIVAGRI project, S.L.E.C.I. irrigation was plotted against reference drip irrigation and rain-fed control in order to compare soil moisture dynamics across different soil depths (30 cm, 60 cm, and 90 cm), irrigation water use, cherry fruit quality traits and yield, and irrigation water productivity (IWP). The data, collected between 2021 and 2023 at the Fruit Growing Institute–Plovdiv test site, reveals that S.L.E.C.I. system demonstrates a clear robustness from short-term climate fluctuations, maintaining root-zone moisture with greater consistency across depths. This contrasts with higher climate dependency observed in the reference variants. The average water productivity of S.L.E.C.I. irrigation is more than 12 times higher compared with the average IWP for drip irrigation. Probably, the superior ratio stems from two factors: first, S.L.E.C.I. delivered only the water that root tension demanded, and second, there is almost no loss of water to evaporation or deep percolation. Statistical analysis confirms that S.L.E.C.I. reduces variability within the crop, delivering significant improvements in both productivity and uniformity, essential traits for high-value commercial fruit production. Despite facing challenges, S.L.E.C.I. remains a promising sustainable irrigation technology, supporting efficient resource utilization while reducing environmental impact.

## 1. Introduction

Increasing water scarcity and climate change present significant challenges to global agricultural production [1]. In regions experiencing prolonged droughts, unreliable rainfall, and elevated temperatures, flexible and sustainable irrigation strategies are essential to ensure crop yield and quality [2,3,4]. In addition, worsened water availability conditions caused by global warming evoke the attention of the scientists to the efficiency of the water use by crops [5]. Therefore, innovations for saving water in irrigated agriculture and thereby improving water use efficiency are of paramount importance in water-scarce regions [6].

Sweet cherry trees (*Prunus avium* L.) are sensitive to water deficits during critical phenological stages, such as flowering, fruit set, and ripening [7,8,9]. The need for water decreases after harvesting and then increases again after the continued growth of the shoots [10]. In order to secure productivity and ensure fruit quality, they require precise water management [11]. Given the high water content in the cherry fruits and their susceptibility to stress-induced quality defects [12,13,14], optimal soil moisture conditions are essential throughout the vegetation. Conventional surface and sub-surface pressurized irrigation systems, including drip irrigation, are available and widely used in commercial orchards for water-efficient production [15]. However, even in these methods exhibit some limitations associated with water distribution, evaporation losses in the topsoil, and dependence on constant energy sources [15,16,17]. Furthermore, with increasing interest in climate-resilient horticultural practices, the integration of passive or low-cost drip irrigation technologies in cherry orchards may become an essential and sustainable solution for water-scarce regions.

The Self-regulating Low-Energy Clay-based Irrigation (S.L.E.C.I.) system represents a novel approach to micro-irrigation [18]. Developed at the Wismar University of Applied Sciences, S.L.E.C.I. utilizes cylindrical, microporous clay emitters buried at root depth and connected via 6 mm tubing to a gravity-fed water source. Water is released into the rhizosphere only when the soil moisture tension exceeds that of the clay emitter, enabling precise, self-regulated delivery without the need for sensors, timers, or electricity.

Much of the population in sub-Saharan Africa make their income from rainfed agriculture and depend to a larger extent on smallholder, subsistence agriculture [19]. Early trials of S.L.E.C.I. with vegetable crops have demonstrated promising results. However, experimental data on the performance of S.L.E.C.I. in temperate tree crops remains sparse.

The present study aims to evaluate the agronomic performance of the S.L.E.C.I. irrigation system in a temperate sweet cherry orchard and compare it to surface drip irrigation and rainfed reference treatment.

## 2. Results

### 2.1. Soil Moisture and Water Consumption

**Figure 1:**
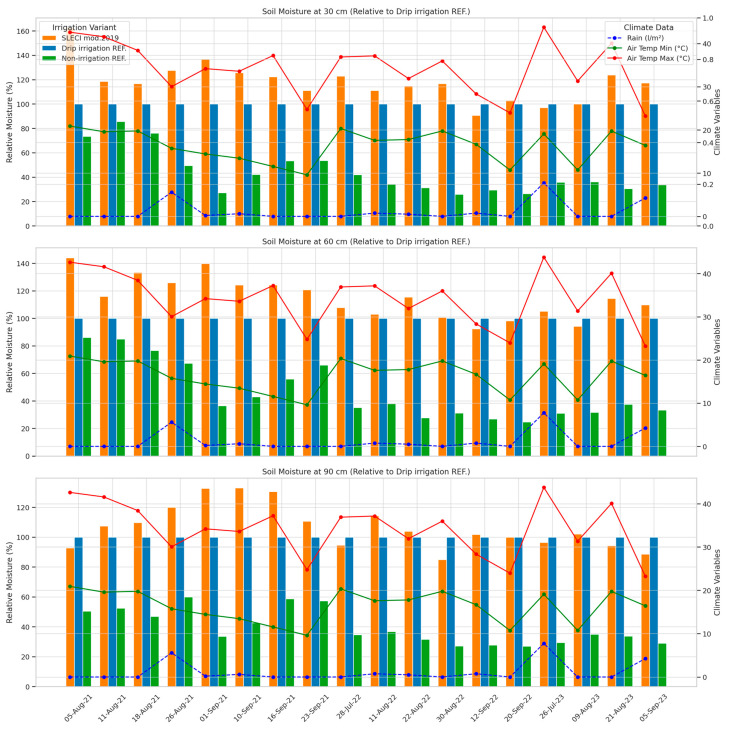
Comparative soil moisture response of irrigation systems with rainfall and temperature trends.

Sweet cherry trees are susceptible to soil water deficit at different stages of fruit development [20], therefore, monitoring of soil moisture and analysing the results is an important tool for determining the soil moisture dynamics during the season, as well as establishing an appropriate irrigation regime [21]. The soil moisture dynamics across different soil depths (30 cm, 60 cm, and 90 cm) for SLECI modification 2019 (SLECI) compared to drip irrigation reference, and non-irrigation treatment for three consecutive years is presented on Figure 1. Soil moisture percentages are represented relative to the drip irrigation reference, which is set as the 100%. Climate variables, including rainfall and air temperature (minimum and maximum), are plotted alongside moisture data, providing critical context for interpreting irrigation efficiency under environmental influences.

Trends across the charts reveal clear distinctions in soil moisture content and distribution among the two irrigation methods and the control. SLECI mod.2019 consistently maintains higher relative soil moisture levels than the non-irrigation variant, and frequently matches or even surpasses drip irrigation reference, particularly at depths of 60 cm and 90 cm. This is especially notable on days characterized by high temperatures and minimal rainfall, emphasizing SLECI’s ability to sustain root-zone moist under climatic stress. The consistency and depth of soil moisture content reinforce the effectiveness of the SLECI system, particularly when drip irrigation struggles to maintain adequate moisture beyond the surface layer. In the results reported in Technical Report on SLECI pilot sites installed within the DIVAGRI Project (2025) [22], SLECI consistently maintained more stable soil water content with smaller fluctuations compared to the surface drip irrigation, as well as with the sub-surface drip irrigation. For example, in lavender and rosemary experiments, the highest oscillation is recorded in the treatment with conventional drip irrigation, followed by the treatment with sub-surface drip irrigation, while the smallest oscillations are recorded in the SLECI treatment. In moringa and cowpea experimental sites, SLECI at 0.3 m depth provided stability in all seasons, and similar results were recorded in sub-surface drip irrigation, in maize experiment, sub-surface SLECI irrigation at 0–15 and 15–30 cm show higher soil moisture content, following by surface drip irrigation and surface SLECI irrigation. A similar condition was observed in the 15–30 cm soil layer, with minor changes after 49 days of sowing, where the surface SLECI treatment showed slightly better moisture content compared to the drip irrigation. Furthermore, better soil water oscillations are recorded in pepper site with SLECI compared to surface drip irrigation.

SLECI’s self-regulating, clay-based micro-irrigation system is designed to respond to plant root demand, delivering water only when suction forces from drying soil trigger release [18,23,24]. This principle is visible in the chart’s consistent moisture values, which remain stable despite fluctuations in weather conditions. Unlike drip irrigation, which can lead to topsoil overwatering and uneven moisture profiles, SLECI demonstrates smoother, deeper penetration with minimal evaporation losses. This aligns with earlier research indicating that conventional surface drip irrigation results in less efficient water use, particularly in hot summer conditions where most of the water is lost from the surface. In contrast, SLECI’s subsurface design minimizes such losses, contributing to both sustainability and plant health.

At 30 cm depth, SLECI occasionally shows slightly elevated moisture levels com-pared to Surface drip irrigation REF., though not excessively so. Rather than indicating over-saturation, these levels are likely to reflect a stable water supply regulated by soil demand. Given that SLECI is passive and self-adjusting, it does not oversupply water; instead, it maintains equilibrium, avoiding both drought stress and waterlogging. This optimal moisture range supports healthy root development, especially for young trees whose roots are more active in the upper layers. On the other hand, the results from other investigations showed initially lower germination and plant emergence under SLECI system installed at 0.3 m depth compared with drip irrigation, suggesting practice to wet the soil in SLECI systems a few days before sowing/planting annual crops, or using some other technique until plant developed proper root system. Such problems were not observed in moringa tree field experiment (Technical Report on SLECI pilot sites installed within the DIVAGRI Project (2025) [22]. According to Agbesi et al. [25], the burial depth had a minimal effect on the emitter discharge but notably affected the advancement and time at which wetting fronts reached the soil surface and bottom boundaries. Operating the SLECI emitter at a higher operating pressure head and shallower burial depth could degrade irrigation water application and water use efficiency, reported same authors. Anyhow, future research should expand to other soil textures, rooting depths and clay dripper installation distance (in the row and in soil depth) enabling the optimal design of the SLECI system for a wider range of crops and environmental conditions. In another study with sweet cherry irrigated with drip system with different frequency, soil moisture content (0 to 20 cm depth) during the growing season was often higher in soils that received high-frequency irrigation (HFI) compared with low-frequency irrigation (LFI). HFI and LFI received the same amount of water, but water was applied four times daily in the HFI treatment but every other day in the LFI treatment [26].

At 60 cm depth, the chart highlights one of SLECI’s greatest strengths: maintaining consistently higher moisture levels compared to both drip irrigation and the non-irrigation variant. Drip irrigation’s performance noticeably drops at this depth, where only a portion of applied water reaches the mid-root zone. SLECI, by contrast, maintains effective moisture here, supporting sustained root uptake and reducing stress from dry periods.

At 90 cm, SLECI continues to outperform the non-irrigation variant and often matches or slightly exceeds the drip irrigation reference. This suggests that although the water delivery efficiency naturally tapers with depth, SLECI still ensures some downward movement of moisture, benefiting deeper root systems. This is particularly important in perennial crops like fruit trees, where deep roots contribute significantly to water uptake. The ability to moist these layers without excess water input demonstrates SLECI’s hydraulic efficiency.

The inclusion of rainfall and temperature data in the chart (Figure 1) provides further in-sights. On days with no rainfall and high air temperatures (above 35 °C), SLECI maintains stable levels, confirming that its delivery mechanism is largely unaffected by surface weather events. This is a key feature: because it is demand-driven and subsurface, SLECI minimizes the influence of external variability. It operates silently in the background, responding only when the root zone calls for water.

SLECI, meanwhile, provides a slow, steady supply that eliminates peaks and troughs. This stability is exactly what fruit trees and other deep-rooting perennials need, reducing stress cycles and encouraging more uniform growth.

The chart indicates no operational failure or significant weakness in the SLECI system. The only observation is that the moisture at 90 cm, although consistently better than the control, is not significantly higher although SLECI tubes are usually installed at a depth of 40–50 cm. Nevertheless, the trend toward deeper soil moist demonstrates the system’s functional reach. The slight decrease in moisture at greater depths reflects natural limits in soil hydraulic conductivity and installation depth, rather than a system failure The SLECI system performs differently in different soil types with different hydraulic characteristics which might influence water availability for plants’ uptake [23]. In study where subsurface irrigation with ceramic emitter’s (SICE) buried at a depth of 40 cm was compared with sub-surface drip irrigation (SDI) in apple trees, the variations in soil water contents for SICE were smaller than those for SDI. Therefore, SICE significantly enhanced yield through its ability to save water and in-crease soil temperature for apple trees [27].

**Figure 2:**
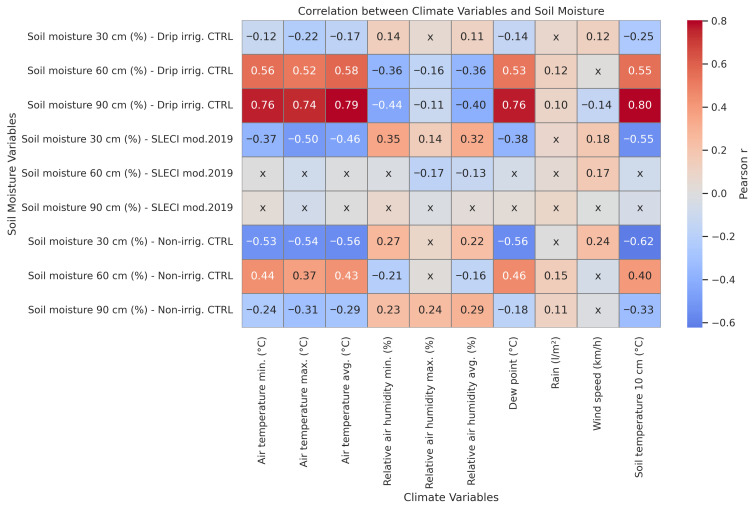
Correlation heatmap-Pearson correlation coefficients between climate variables and soil moisture across irrigation treatments and depths. Non-significant correlations (*p* ≥ 0.05) are marked with “x”. Colour scale indicates direction and strength of correlation.

The correlation analysis between climate variables and soil moisture across the three irrigation variants (SLECI modification 2019, drip irrigation reference, and non-irrigation variant) provides critical insight into how each system responds to environmental conditions at different soil depths. Figure 2 presents Pearson correlation coefficients between daily climate variables and soil moisture across the three irrigation strategies and depths. Significant correlations (*p* < 0.05) are shown numerically, while non-significant relationships are marked with “x”.

Air temperature (minimum, maximum, and average) demonstrated a strong negative correlation with soil moisture in the drip irrigation and non-irrigation variants, particularly at 30 cm. This reflects the vulnerability of surface moisture to evaporation under high-temperature conditions. In contrast, the SLECI mod.2019 variant exhibited non-significant correlations, especially at 60 and 90 cm depths, indicating its capacity to maintain consistent subsurface moisture regardless of thermal stress. This stability supports the self-regulating nature of the SLECI system, which operates based on plant-driven soil suction rather than external environmental triggers [20].

Rainfall and wind speed exhibited weaker and more variable associations, often non-significant. These patterns highlight the differential climate sensitivity of soil moisture under each irrigation strategy and support the observed performance of SLECI in maintaining subsurface moisture stability. This highlights limited water infiltration and retention without active or subsurface irrigation. In SLECI, rainfall had insignificant correlation across all depths, confirming the system’s independence from precipitation variability.

Again, SLECI’s correlations to soil temperature at 10 cm were consistently lower, suggesting effective buffering from surface conditions.

In essence, the SLECI system demonstrates a clear decoupling from short-term climate fluctuations, maintaining root-zone moisture with greater consistency across depths. This contrasts with the higher climate dependency observed in drip irrigation and non-irrigation variants.

### 2.2. Irrigation Water Use in Sweet Cherry by Treatments and Years

The results about irrigation water use by treatments during the period 2021–2023 are presented below in Table 1.

Irrigation records in Table 1 show a different use of water during the season. The SLECI system showed very low irrigation water use across all years, with applied water ranging from 70.3 m^3^/ha in 2021 to 226.5 m^3^/ha in 2022, and a three-year average of 157.2 m^3^/ha. When rainfall was included, the total water ranged from 171.2 m^3^/ha (2021) to 422.6 m^3^/ha (2022), averaging 314.3 m^3^/ha. This indicates that SLECI is a highly water-efficient system, supplying only small but steady rates of water [18,28]. Drip irrigation had by far the highest irrigation water use in all three years.

Applied irrigation water during the season ranged from 960.5 m^3^/ha in 2021 up to 3161.6 m^3^/ha in 2022, with a three-year average of 2183.3 m^3^/ha. Including rainfall, totals were between 1061.3 m^3^/ha (2021) and 3357.7 m^3^/ha (2022), averaging 2340.4 m^3^/ha. This means drip irrigation applied over 14 times more water than SLECI on average, reflecting very high input requirements. Similar trends of high input needs in drip systems compared to alternative technologies have been noted in wolfberry [29] and apple fruits [27]. In non-irrigated treatments, no irrigation was applied, and total water use reflected only rainfall contribution: 100.9 m^3^/ha in 2021, 196.1 m^3^/ha in 2022, and 174.3 m^3^/ha in 2023, with a three-year average of 157.1 m^3^/ha. The results show strong dependence on this treatment with rainfall, which is both variable and insufficient to meet crop water needs reliably. All treatments show higher values in 2022, suggesting longer irrigation schedules with more demanding climatic conditions. The difference between irrigation methods is most evident in 2022, when drip irrigation volumes were extremely high while SLECI remained relatively low. Our results correspond with those presented in Technical Report on SLECI Pilot Sites Installed within the DIVAGRI Project (2025) [22], where SLECI demonstrate lesser irrigation water use compared with drip irrigation across multiple crops (lavender, rosemary, moringa, cowpea, maize, gem squash, and tomato). According to the same report, while drip irrigation guarantees abundant water delivery, it does so at the cost of very high input water volumes. In contrast, SLECI offers a balance between reduced water application and maintained crop performance, making it particularly suitable for areas facing water scarcity or high irrigation costs.

### 2.3. Sweet Cherry Yield by Irrigation Treatments and Year

Many scientific literatures demonstrate that carefully managed irrigation is a critical factor in maximizing sweet cherry yield and fruit quality. Providing adequate water during key phenological stages significantly enhances fruit size, weight, and overall crop volume. An adequate water supply is imperative during the flowering and fruit development phenology stages, as well as in the post-harvest period [30]. Although sweet cherries are susceptible to overwatering, which can lead to undesirable effects such as fruit cracking. Irrigated sweet cherries compared to production under rain feed conditions, as well as strategic irrigation carried out with appropriate techniques, affect the productivity and quality of sweet [18,26,31,32,33].

Various irrigation strategies have been explored, ranging from full replacement irrigation—matching all water lost to evapotranspiration—to advanced deficit irrigation methods like Regulated Deficit Irrigation (RDI) and Postharvest Deficit Irrigation (PDI). A recent global meta-analysis encompassing apples, peaches, and sweet cherries confirms that irrigation scheduling is paramount for optimizing yield and water use ef-efficiency [31]. Moreover, irrigation management plays a vital role beyond the growing season. Postharvest deficit irrigation research highlights that maintaining adequate soil moisture after harvest and maintain fruit yield at similar levels to fully irrigated trees [34]. Applying moderate water deficits postharvest can conserve and does not affect negatively, fruit quality and yield responses of cherry trees [35].

In addition to yield improvements, irrigation technique influences fruit marketability and quality. Specifically for sweet cherries, high-frequency irrigation—delivering smaller volumes of water more often—has been shown to sustain more consistent root zone moisture, thereby increasing fruit size and yield com-pared to less frequent, heavier applications [26]. This approach also contributes to enhanced tree vigour, as evidenced by greater trunk cross-sectional area and biomass accumulation. In another research study, two irrigation systems of double-lateral drip line and micro-sprinklers in-row ground cover systems of wheat straw mulch and the control (no ground) were evaluated. Sweet cherry, double-lateral drip irrigation increased marketable fruit by 8.6% on average relative to micro-sprinklers [32]. According to Yin et al. [33], fruit yield and fruit quality including firmness, colour, and size, did not differ regardless of irrigation or ground cover system. Drip irrigation increased marketable fruit by 7% to 12% via reducing fruit surface pitting and bruising compared with micro-sprinkler. Deficit irrigation regimes have been shown to reduce rain-induced fruit cracking significantly, increasing the proportion of marketable cherries [36]. By irrigating the trees according to the soil water potential, it was possible to reduce the pre-harvest irrigation by 24% on average over the years compared to daily-irrigated trees, without any negative effect on yield and fruit quality [37].

Emerging technologies like Self-Regulated Low-Energy Clay Irrigation (SLECI), a subsurface system using clay emitters that self-regulate based on soil suction force, offer promising alternatives for sustainable water use. Preliminary studies on SLECI in sweet cherry plantations show potential for maintaining yields with reduced energy and water inputs compared to traditional drip systems, though long-term data is still developing [18].

Finally, in this paper the results of sweet cherry yields are presented obtained from our experiment concluded with SLECI irrigation technology compared with surface drip irrigation and non-irrigated treatment. The field experiment with sweet cherry was established in 2019, and first fruits are recorded in 2021. However, this cannot be considered as yield because just several fruits per tree were established. The first yielding year was 2022. 2023 was first year with significant yield for the young orchards and in 2023 the sweet cherry trees still did not reach their full yielding potential. However, the results presented in Table 2 for sweet cherry yields show different yields for the young sweet cherry tree as a result of applied irrigation methods.

The results demonstrate a clear positive impact of both self-regulating low energy clay-based irrigation (SLECI) and drip irrigation on sweet cherry yield compared to the non-irrigated control. Across the years 2021 to 2023, irrigated treatments consistently produced significantly higher yields, with average yields of 1147.24 kg/ha under SLECI and 1233.95 kg/ha under drip irrigation, whereas non-irrigated trees yielded only 0.46 kg/tree (306.82 kg/ha). These findings align with well-established research showing that irrigation greatly enhances cherry productivity by improving fruit size, weight, and overall yield potential [26]. Notably, drip irrigation achieved higher average yields than SLECI, probably due to supplying more water to deeper soil layers. However, the relatively small difference in yield suggests that SLECI offers a comparable alternative with potential advantages in energy and resource efficiency, consistent with emerging research on innovative irrigation systems for sustainable fruit production [18]. Furthermore, in the experimental studies reported in the Technical Report on SLECI Pilot Sites Installed within the DIVAGRI Project (2025) [22], combined results were obtained under different irrigation techniques and crops yields. In four of the six experimental sites SLECI matched or exceeded drip yields up to 78% in tomato to only 7% in maize, than almost equal in moringa and gem-squash, showing that deep or perennial root systems readily adapt to self-regulated trickle supply. The shallow-ooted cowpea lost 18% biomass because SLECI emitters were buried beyond its effective rooting depth, while first year rosemary and lavender showed better yielding in drip than in SLECI irrigation treatments. Both issues can be solved by shallower placement of SLECI lines in the first season by pairing SLECI with limited surface sprinkling or drip during crop establishment. Therefore, with modest agronomic fine--tuning, the technology is compatible with a far broader crop portfolio. In other research investigations, the highest yield in SLECI irrigation was recorded in bell pepper treatment under a burying depth of 5 cm which is 8.6% and 63.9% more compared to 10 cm and 15 cm [23]. In study with apple trees, where subsurface irrigation with ceramic emitters (SICE) buried at a depth of 40 cm was compared with sub-surface drip irrigation (SDI), results shown that SICE significantly improved new shoot length and yield by 15.9% and 7.6% [27]. Han et al. [29] reported that the SICE system outperformed surface drip irrigation and subsurface drip irrigation in terms of wolfberry yields, with average increases of 8.0% and 2.3%, respectively.

In summary, the scientific evidence overwhelmingly supports that irrigation is indispensable for achieving high yields and superior fruit quality in sweet cherry production. Optimal irrigation approaches with innovative systems like SLECI should be tailored to local climatic, edaphic, and water resource conditions to maximize yield, as well as to improve water use efficiency/irrigation water productivity and sustainability.

### 2.4. Irrigation Water Productivity in Sweet Cherry Under SLECI and Drip Irrigation Techniques

WP generally serves as a key performance indicator for evaluating the effectiveness of irrigation water management [38], while irrigation water productivity (IWP) refers to the efficiency with which irrigation water is used to produce crop yields. Unlike crop water productivity or water use efficiency (WUE), which may include total water used (evapotranspiration) by plants [39,40], IWP focuses specifically on applied irrigation water. According to Gang et al. [41], IWP is the ratio of crop yield obtained per unit of irrigation water use, reflecting the management level of irrigation and crops technologies. It is often expressed to emphasize maximizing crop production under water constraints conditions [42], but also, to compare irrigation techniques or irrigation methods used in agricultural production [22]. Cetin et al. [43] in their investigations give focus on irrigation water productivity, because the amount of irrigation water used in a farm and/or irrigation scheme is more important for farmers and irrigation authorities. Finally, the IWP is important for maintaining agricultural productivity, particularly to the expected climate change, thus knowing factors that influence IWP and development of the strategies for improving IWP are important scientific topics.

Lacerda and Oliveira [44], reported that irrigation is a highly attractive agricultural technology, but it is only profitable and sustainable when properly performed, through techniques that maximize efficiency, promoting operational costs reduction and environmental impacts. In agriculture, it is important to produce more with less water because water is a limiting factor in many parts of the world. Ali & Talukder [42] reported that water is an economic good, because we have to pay for it, and in many cases, we also have to pay enormous environmental costs. Therefore, increasing crop yields per unit of water used is crucial for sustainable agriculture amid global water scarcity, where agriculture consumes around 70% of freshwater [43,45,46]. In this context, just with switching from micro-sprinklers to double line drip irrigation can remarkably reduce irrigation water use in sweet cherry up to 54.3% [32]. Similarly, single-lateral drip irrigation has the potential to be a viable alternate irrigation system for sweet cherry production in comparison with micro-sprinklers and to increase water use efficiency from 167 to 234% [33].

As was mentioned above, irrigation-water productivity (IWP) answers to the most asking question: “How many kilograms of marketable yield did each cubic meter of water?”. In our case, the results for IWP in sweet cherry under sub-surface SLECI and surface drip irrigation are presented in Table 3. Considering the methodology for determining IWP, our results for this parameter are based only for sub-surface SLECI and surface drip irrigated treatments. Also, since the first comparable sweet cherry yields were recorded in 2022 and 2023, the results processed for the IWP are analysed only for these two years of investigation.

The results obtained for IWP in our investigation show that younger sweet cherry trees reacted much better in sub-surface SLECI irrigation compared to surface drip irrigation. The irrigation water productivity (IWP) in sweet cherry ranges from 0.15 kg/m^3^ under surface drip irrigation up to 3 kg/m^3^ for SLECI treatment in 2022. In 2023, the sub-surface drip irrigation treatment reached 0.44 kg/m^3^, while SLECI irrigation treatment de-livered 9.23 kg/m^3^. The average water productivity in case of SLECI irrigation is more than 12 times higher compared with the average IWP for drip irrigation. Probably, the superior ratio stems from two factors: first, SLECI delivered only the water that root tension demanded, and second, there is almost no loss of water to evaporation or deep percolation. Similar results for improving IWP in SLECI technology are reported in rosemary and lavender herbs, maize, moringa, cowpea, tomato and gem squash under semi-arid savannah, humid tropics and saline desert soils compared with the most practical and very commonly used surface or subsurface drip irrigation technique [22]. In context of productive use of irrigation water, results from the same report showed that sub surface drip is more productive than surface drip irrigation, mainly due to advantage of drip line installation in the soil in comparison with the surface drip irrigation treatment. Furthermore, Osei et al. [23] in their investigation with bell pepper crops presented better water use efficiency of SLECI irrigation lines in burying depth of 10 cm compared with the 5 and 10 cm. In another research study with innovative irrigation technologies, the subsurface irrigation with ceramic emitters (SICE) buried at a depth of 40 cm shown significantly improved WUE and IWUE by 14.8% and 6.5% compared to subsurface drip irrigated apples [27]. Furthermore, in research study with wolfberry, same technology (SICE) showed higher water use efficiency, with 14.6% and 4.5% increases compared to drip irrigation and subsurface drip irrigation, respectively [29].

Finally, future research should expand to other soil textures and rooting depths, enabling the optimal design of the SLECI system for a wider range of crops and environmental conditions [18,22,24,25].

### 2.5. Fruits Quality Traits and Yield

**Figure 3:**
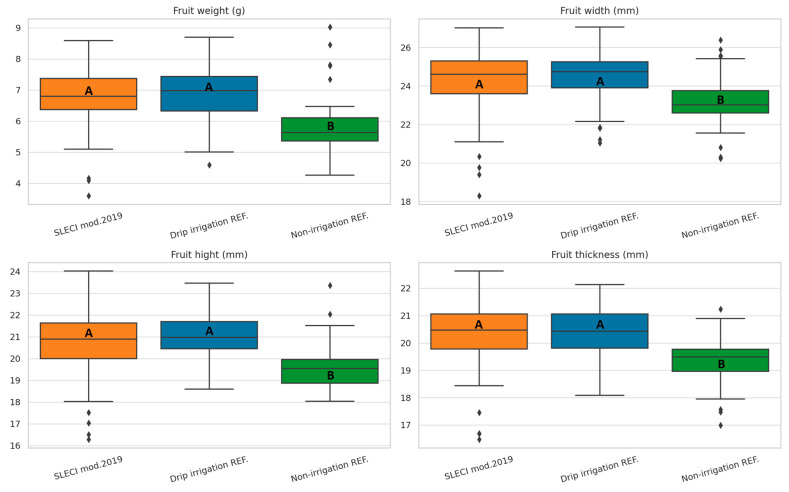
Effects of irrigation strategy on fruit size and weight characteristics, statistical differences among treatments confirmed by ANOVA (*p* < 0.001) and LSD post-hoc test (α = 0.05). Different letters indicate statistically significant differences between treatments based on LSD post-hoc test (α = 0.05).

Significant differences in fruit biometric traits (fruit weight, width, height, and thickness) among irrigation variants are evident from Figure 3. Statistical analysis confirms these differences are significant (ANOVA *p* < 0.001 for all traits), with post-hoc LSD tests revealing distinct groupings across treatments.

For fruit weight, SLECI modification 2019 exhibits similar means to the drip irrigation reference, and significantly greater non-irrigation variant. The narrow interquartile range and dense clustering of values reflect consistent performance and low sample variation, indicative of stable water availability throughout the growing period. Drip irrigation displays moderate variation, while non-irrigation variant shows a broader spread and more outliers, highlighting uneven growth due to water stress.

Similar patterns are evident in fruit width and thickness, where SLECI again surpasses with the highest means and tightest distributions. Fruit height follows the same trend, though with slightly less separation between SLECI and Drip irrigation. The consistently larger dimensions under SLECI suggest enhanced cell expansion and fruit development, likely a result of its ability to maintain optimal and uniform soil moisture at the root zone through self-regulation.

In contrast, the variant where no irrigation was applied consistently produces the smallest and most variable fruits, underscoring the impact of water limitations on fruit development.

The statistical analysis confirms that SLECI not only increases fruit size but also reduces variability within the crop, delivering statistically significant improvements in both productivity and uniformity, essential traits for high-value commercial fruit production.

**Figure 4:**
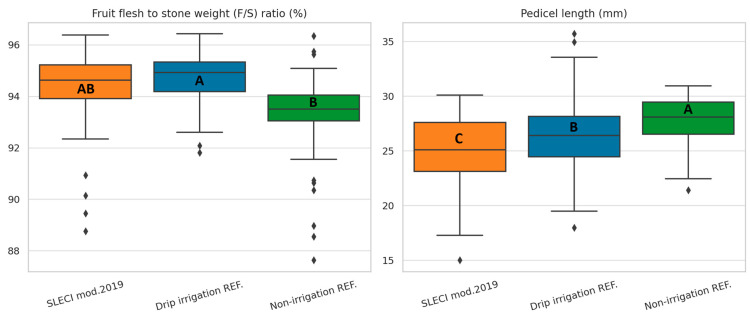
Effects of irrigation strategy on flesh-to-stone (F/S) ratio and pedicel length, statistical differences among treatments confirmed by ANOVA (*p* < 0.001) and LSD post-hoc test (α = 0.05). Different letters indicate statistically significant differences between treatments based on LSD post-hoc test (α = 0.05).

For flesh-to-stone ratio, drip irrigation showed slightly higher means than SLECI, though not significantly different. Non-irrigation again lagged with lower, more variable ratios (Figure 4).

The non-irrigation variant exhibited the longest pedicel, significantly higher values than both SLECI mod.2019 and drip irrigation reference. Variation within SLECI samples was also minimal, reflecting consistent development under stable moisture conditions.

## 3. Discussion

### 3.1. S.L.E.C.I. Origin and Principles

The “S.L.E.C.I.” technology (Self-regulating, Low Energy, Clay based Irrigation) is an innovative clay-based micro-irrigation system developed by Prof. Dr. Harald Hansmann at the Institute for Polymer- and Produktionstechnologies (IPT) and patented by the Wismar University of Applied Sciences (HSW) in Germany (patent number: DE 102019005311.7).

The core of the S.L.E.C.I. systems are the clay emitters, which comprise of a clay tube with micro-pores tailored to specific conditions including crop type and soil type. Additionally, it features a preassembled connector that interfaces with 6 mm conventional irrigation tubes on both ends. Depending on crop and field characteristics, S.L.E.C.I. emitters differ by size, content (clay recipe), and layout can vary from horizontal to vertical.

In this technology, water is transferred to the soil via cylindrical clay emitters connected to each other by 6 mm pipes and buried in the soil near the roots of the plants. This system, consisting of clay elements and pipes, is then connected to a water source via connectors, and water moves via gravity. The SLECI system can operate without electricity. Water is delivered to the clay tube’s surface through permeation, driven by soil suction tension. The clay body’s geometry and hydraulic conductivity must be aligned for effective water release. Hydraulic conductivity depends on the clay’s capillary pore structure, influenced by its composition and firing (manufacturing) conditions. Capillarity creates a suction tension based on pore size distribution, which opposes the soil’s suction tension. Water can be pressurized directly from a tank. The hydraulic conductivity of the clay emitter allows the surrounding soil to draw water from the system through the clay, due to its suction tension. Ideally, the amount of water permeating through the clay element matches the amount evaporated by the plant via its leaf surface. However, losses from deep percolation and evaporation at the soil surface must also be accounted for. The soil acts as a buffer storage for water, with a time delay determined by the speed at which water permeates between the clay body and the plant roots. Moreover, the mineral composition of the soil plays an additional role in the irrigation process [18].

S.L.E.C.I. is very simple in concept, flexible in installation and therefore fulfils all demands of irrigation systems in rural environments. It is a self-regulating subsurface irrigation technique, without the need for external water management systems and measurement of the actual soil humidity. It is water saving, a low-energy system (uses gravity to deliver water to emitters), operates sustainably and is environmentally advantageous (no risk of over-irrigation and groundwater pollution, precise fertilisation dosage through the water etc.).

### 3.2. S.L.E.C.I. Development and Testing

In early initial tests, S.L.E.C.I. has provided convincing results with moringa trees, mango and cucumbers in several countries, mainly focused in Africa [28].

In 2018, an experimental cherry orchard was established in Plovdiv, Bulgaria within the framework of project No. 01DS19037: “Transnational Partnership for Implementation of Micro-Irrigation Technology in Bulgaria and North Macedonia” (duration 1 June 2019 to 31 December 2020) [47], and the research was continued in the test field as part of a project under the EU Framework Programme for Research and Innovation “Horizon 2020” with reference number 101,000,348–DIVAGRI and title “Revenue diversification pathways in Africa through bio-based and circular agricultural innovations” (duration 1 June 2021 to 31 May 2025) [19].

The DIVAGRI project proposes a wide range of bio-based innovative solutions adapted to specific conditions in target countries [48]. The goal is to enhance the productivity, income, and economic opportunities of subsistence and smallholder farmers in arid and semi-arid Sub-Saharan Africa by using innovative bio-based solutions. These solutions aim to improve agricultural production, diversify crops, increase added value, create sustainability, and generate new local economic opportunities in an environmentally friendly manner over the long term. DIVAGRI has screened existing bio-based solutions and selected a set of technologies to transfer from European project partners to pilots and demonstration sites managed by African project partners including leading R&D institutes. These technologies improve farming inputs, diversify food production, and enable bio-based products from agricultural by-products and waste. Biochar, biogas, and biorefineries offer both stationary and mobile applications, fostering new business models. Selected solutions include diverse crop systems, mobile biorefineries, optimized biochar and biogas processes, the innovative S.L.E.C.I. clay-based micro-irrigation system, vegetation-enhanced solar desalination greenhouses, and multifunctional wetlands [49].

The parallel piloting of S.L.E.C.I. irrigation systems among project partners reveals significant potential for improving water and fertilizer efficiency in crop production, particularly in water-scarce regions. Research findings indicate that optimal SLECI burying depth plays a crucial role in influencing crop yield, water use efficiency (WUE), and fertilizer use efficiency (FUE) [18,23].

In Bell pepper (*Capsicum annuum* L.) cultivation, a 10 cm burying depth provided the best balance of moisture retention and nutrient availability, resulting in superior WUE (0.1435 kg/L) and FUE (5980 kg/kg) while reducing deep percolation losses. The highest yield (0.8772 t/ha) was achieved with 80% recommended fertilizer dosage (RAD), which improved nutrient uptake efficiency without excessive application. Additionally, the interaction of 10 cm burying depth and 80% RAD produced the best overall performance, maximizing yield (1187.7 t/ha), WUE (0.214 kg/L), and FUE (8908 kg/kg) [23].

Beyond the DIVAGRI project, SLECI is also being trialled in the Mediterranean-focused MED-WET initiative, where it has demonstrated significant water savings in citrus, vine grapes, and stone fruit crops across Malta, Morocco, and Portugal. These findings support its adaptability to diverse agroecological conditions and reinforce its potential for broader deployment.

### 3.3. S.L.E.C.I. Alternatives and Competition

Self-Regulating Low-Energy Clay-Based Irrigation (S.L.E.C.I.) is an emerging subsurface irrigation system that utilizes porous clay emitters to deliver water directly to plant roots. While S.L.E.C.I. exhibits significant advantages in water conservation and energy efficiency [18], it faces competition from various clay-based and subsurface irrigation technologies, including Subsurface Irrigation with Ceramic Emitters (SICE) [50], Ceramic-Patch-Type Subsurface Drip Irrigation Line (CP-SDIL) [51], clay pot irrigation, and porous clay pipe systems. SICE employs microporous ceramic emitters, delivering water with enhanced uniformity at lower pressure heads than conventional drip irrigation. CP-SDIL integrates ceramic patches in subsurface drip lines, improving emitter durability and reducing clogging risks. Traditional clay pot irrigation and porous clay pipe systems provide efficient soil moisture retention but remain labour-intensive and challenging to scale [24].

The literature review reveals [24] that S.L.E.C.I. outperforms competitors in self-regulation, adjusting water discharge according to soil moisture levels, reducing deep percolation losses, and preventing overwatering. Unlike traditional clay pot systems, S.L.E.C.I. integrates modular clay emitters within a pipeline structure, facilitating installation and adaptability across different field conditions. Additionally, its low energy requirement—operating purely on gravitational pressure—renders it superior to SICE and CP-SDIL, which rely on external pressure regulation. Despite its advantages, S.L.E.C.I. exhibits high initial production costs, particularly in the fabrication of fired clay emitters, restricting scalability. Compared to SICE, its hydraulic conductivity is lower, which may impact soil wetting uniformity in coarse-textured soils. Additionally, installation complexity necessitates precision to ensure optimal water delivery.

### 3.4. S.L.E.C.I. Future Development and Possible Applications

Despite its efficiency, SLECI faces challenges, including high initial production costs and installation complexity. Compared to other irrigation systems, its hydraulic conductivity is lower, affecting soil wetting uniformity in certain conditions. The underground layout of the system, although allowing soil cultivation at 20–25 cm depth without damaging the components of the system, hinders inspection and replacement of clay emitters. On several occasion damage to the 6 mm connecting tubes by rodents have been observed. While such incidents are rare, they reveal a potential weakness given the diversity of environments and crops within the DIVAGRI and MED-WET projects. Therefore, raising awareness and developing mitigation strategies will be essential for scaling SLECI across varied agroecological zones.

Future research should focus on improving cost-effectiveness and adaptability for large-scale applications. Further development in clay recipes to improve hydraulic conductivity is undergoing and new layouts to facilitate addition or replacements of clogged/broken clay emitters are already being tested.

In Europe, “SLECI” is applicable in the growing fields of urban gardening and landscaping. The system conserves both water and time. It can be implemented for container cultivation of vegetables, flowers, and fruit trees in pots on balconies or green spaces between city buildings, without risking waterlogging and flooding of subterranean premises and basements of residential buildings. In agriculture, it is suitable for small farmers in remote and hard-to-reach areas [52]. The lack of need for electricity, combined with the self-regulating effect, allows practical use of various water sources, tubes and tanks to small reservoirs, with minimal maintenance and low investment costs for building the system.

A valuable direction for future investigation may include a preliminary life cycle assessment (LCA) to evaluate the environmental footprint of SLECI components and compare them with conventional irrigation systems, providing a more comprehensive understanding of its sustainability potential

SLECI remains a promising sustainable irrigation technology, supporting efficient resource utilization while reducing environmental impact.

## 4. Materials and Methods

### 4.1. Research Design

Experimental orchard of 60 sweet cherry trees of cultivar ‘Bigarreau Burlat’ grafted on *P. mahaleb* seedling was planted on 6 March 2019. Irrigation systems were installed on 11 July 2019 with three variants—the new micro-irrigation with clay tubes S.L.E.C.I. and as controls-variants with drip irrigation (60 cm between drippers) and non-irrigation (rain-fed) reference.

### 4.2. Climate and Soil Conditions at the Experimental Site in Plovdiv, Bulgaria

The trial was set in a region with a transitional continental climate, which brings distinct seasonal contrasts. Weather and soil data was collected by Meteobot^®^ Pro automatic weather station for agriculture situated inside the experimental site at the Fruit Growing Institute–Plovdiv.

**Figure 5:**
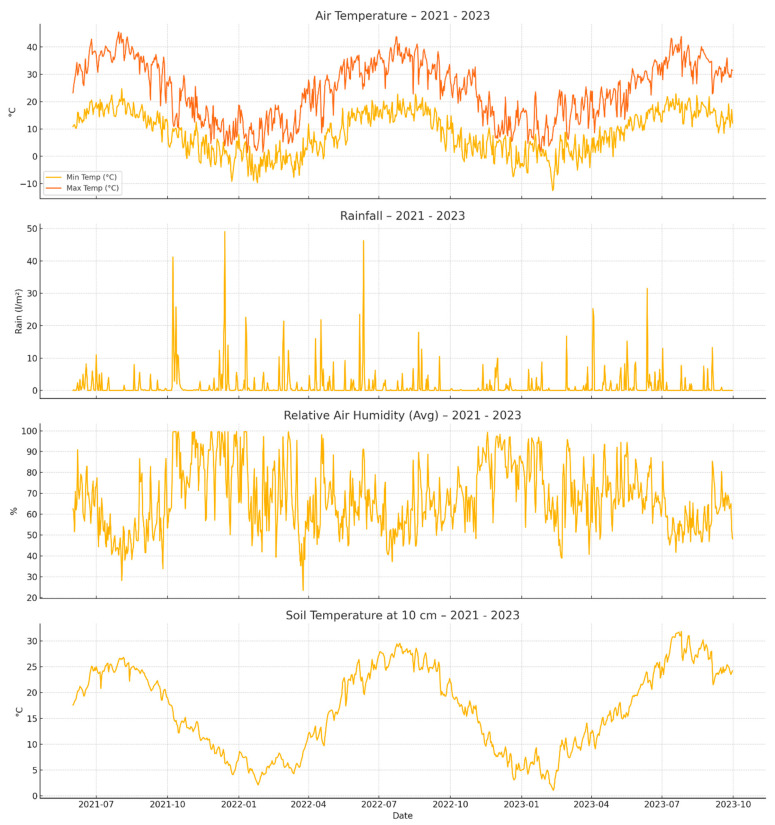
Climate conditions at the experimental site.

The data collected (Figure 5) between 2021 to 2023 reveals a pattern of hot, dry summers and cool, wetter winters—conditions typical for south-central Bulgaria.

During the summer months (June to August), maximum air temperatures frequently exceeded 35 °C, and in several cases even surpassed 40 °C, particularly in July and August of 2022 and 2023. These periods coincided with very limited rainfall and high atmospheric water demand. Rainfall during these months was both sparse and irregular. Extended dry spells, such as those recorded in June through mid-August 2022, presented extreme stress conditions for unirrigated plants.

Soil temperatures at 10 cm depth mirrored the air temperatures, often rising to 30 °C or more during peak summer. At the same time, relative humidity levels dropped below 40–50%, further exacerbating evapotranspiration losses.

In contrast, the cooler months (late autumn to early spring) brought more consistent rainfall and lower temperatures. From October through March, minimum air temperatures often fell below 5 °C, with frost conditions occasionally recorded in the winter. Rainfall increased, particularly during late autumn and early spring, with daily accumulations of up to 25 l/m^2^. Relative humidity during this period regularly exceeded 80%, and soil temperatures dropped to around 5–6 °C by mid-winter.

These seasonal shifts had a direct impact on soil water availability. While the winter rainfall recharged the soil, plant uptake was limited during dormancy. In contrast, the summer months presented a clear demand for consistent and deep irrigation, particularly to support optimal root zone moisture and avoid moisture stress during critical growth phases.

Soil properties of the experimental field were previously analysed in Ss. Cyril and Methodius University of Skopje, North Macedonia [18]. Bar Pressure Plate Extractor 532-100 and Pressure Plate by Ele International were used for analysis of soil water retention [53,54]. The results showed that the soil is very sandy with low water retention (about 8.05% at 0.33 bars and 4.5% at 15 bars) reducing the effectiveness of the SLECI clay-tube micro-irrigation.

### 4.3. Soil Moisture and Water Consumption

Precise measurements were performed using CPN 503DR HYDROPROBE, neutron moisture probe that measures the sub-surface moisture in soil and other materials by use of a probe containing a source of high energy neutrons and a slow (thermal) neutron detector.

Additionally, the dynamics of soil moisture were measured in real-time using Meteobot^®^ Nano with Soil Moisture Sensor SW10 (Prointegra Ltd.; Varna, Bulgaria) placed at every irrigation variant at 30 cm, 60 cm and 90 cm depths.

Water consumption in the different irrigation variants was measured using Axioma QALCOSONIC W1 ultrasonic water meters. Additional manual checks were performed at the water tank for SLECI in periods with low flow rates.

### 4.4. Irrigation Water Productivity Analysis

In agriculture, irrigation water productivity is defined as the ratio between the actual crop yield achieved and the water use, expressed in kg/m^3^ [55,56]. Actually, irrigation-water productivity (IWP) measures how many kilograms of product is obtained from each cubic meter of water and is commonly used method for comparing irrigation technologies that use different volumes and ways of water application in agricultural production. In our analysis, we have used the folowing equation for estimating the IWP.

IWP = Ya/Iwa,(1)

where: IWP is irrigation water productivity in kg/m^3^, Ya is crop yield in kg/ha, while Iwa is irrigation water applied in m^3^/ha.).

### 4.5. Fruits Quality Traits and Yield

First flowering (Apr-2020) and fruiting (Jun-2020) of the trees in the test field occurred in the second vegetation after planting. All variants, including the control, produced flower buds in 2019 and started to bloom late in March 2020. In the first days of April-2020, in the phenological phase “full flowering”, after a series of spring frost incidents, most of the flowers were damaged and the crop was reduced to single fruits. In 2021, during the first year of project DIVAGRI, the trees produced the first typical fruits.

In 2023, the yield was affected by spring frost damage for the second time (Table 2).

Fruit biometric data was measured with Mitutoyo Digimatic Caliper CD-15DAX for fruit size and digital scale Kern EHA 500-2 for fruit weight.

### 4.6. Data Analysis and Processing

Data was subjected to statistical analysis using the software products “R-4.4.2” in combination with “RStudio Desktop 2024.12.0” and installed package “agricolae 1.3-7” [57].

Treatment effects were evaluated using one-way analysis of variance (ANOVA), followed by Least Significant Difference (LSD) tests at a significance level of α = 0.05. For fruit size and weight traits, statistical significance was confirmed at *p* < 0.001.

Pearson correlation analysis was performed to assess relationships between daily climate variables and soil moisture at 30 cm, 60 cm, and 90 cm depths across the three irrigation strategies. Correlation coefficients (r) were calculated, and statistical significance was determined (*p* < 0.05). Non-significant correlations were masked in the heatmap (Figure 2) to improve interpretability.

During the preparation of this manuscript, the authors used Microsoft Copilot^®^ (version current as of April 2025) to assist in refining a custom R script developed and executed in RStudio. The script was originally created by the authors to process and analyse data via “agricolae: Statistical Procedures for Agricultural Research” package, and Copilot was used specifically to enhance the visualization component. The authors have reviewed and edited the output and take full responsibility for the content of this publication.

## 5. Conclusions

The results reinforce the suitability of SLECI for sustainable and climate-resilient agriculture, particularly in regions facing heat stress and irregular rainfall patterns. Its performance supports more stable soil hydration, essential for long-term crop health and water conservation.

## Figures and Tables

**Table 1:** Irrigation water use by treatments and years.

Treatments/Years	Sum of Applied Water in m^3^/ha	Days of Application	Sum of Applied Water with Rain Fall in m^3^/ha
SLECI mod.2019/2021	70.3018 b	72	171.1522 b
SLECI mod.2019/2022	226.4932 b	237	422.6245 b
SLECI mod.2019/2023	174.8074 b	182	349.0611 b
SLECI mod.2019/Average	157.1986 b		314.2771 b
Drip irrigation REF./2021	960.48 a	72	1061.33 a
Drip irrigation REF./2022	3161.58 a	237	3357.711 a
Drip irrigation REF./2023	2427.88 a	182	2602.134 a
Drip irrigation REF./Average	2183.311 a		2340.39 a
Non-irrigation REF./2021	0.00 b	72	100.8504 b
Non-irrigation REF./2022	0.00 b	237	196.1314 b
Non-irrigation REF./2023	0.00 b	182	174.2538 b
Non-irrigation REF./Average (rainfed)	0.00 b		157.0785 b

Different letters indicate statistically significant differences between treatments based on LSD post-hoc test (α = 0.05).

**Table 2:** Effect of irrigation techniques on sweet cherry yield.

Treatments/Years	Average Sweet Cherry Yield in kg/ha	Frost Damage (%)
SLECI mod.2019/2021	Single fruits *	No damage
SLECI mod.2019/2022	680.34 a	No damage
SLECI mod.2019/2023	1614.14 a	35.93 ab
SLECI mod.2019/Average	1147.24 a	
Drip irrigation REF./2021	Single fruits *	No damage
Drip irrigation REF./2022	480.24 a	No damage
Drip irrigation REF./2023	1987.66 a	16.05 b
Drip irrigation REF./Average	1233.95 a	
Non-irrigation REF./2021	Not fruits **	No damage
Non-irrigation REF./2022	306.82 a	No damage
Non-irrigation REF./2023	306.82 b	51.6 a
Non-irrigation REF./Average (rainfed)	306.82 b	

* Beginning of fruiting was in 2021 (planted in March 2019). ** Beginning of fruiting was in 2022 (planted in March 2019). Different letters indicate statistically significant differences between treatments based on LSD post-hoc test (α = 0.05).

**Table 3:** Irrigation water productivity in sweet cherry kg/m^3^.

Irrigation Treatments/Year	Average Sweet Cherry Yield in kg/ha	Water Applied for Irrigation in m^3^/ha	Irrigation Water Productivity in kg/m^3^
SLECI mod.2019/2022	680.34	226.49	3.00
SLECI mod.2019/2023	1614.14	174.81	9.23
SLECI mod.2019/Average	1147.24	200.65	5.72
Drip irrigation REF./2022	480.24	3161.58	0.15
Drip irrigation REF./2023	1987.66	2427.88	0.82
Drip irrigation REF./Average	1233.95	2794.73	0.44
Non-irrigation REF./2022	306.82	0	n/a *
Non-irrigation REF./2023	306.82	0	n/a *
Non-irrigation REF./Average (rainfed)	306.82	0	n/a *

* n/a—not applicable.

## Data Availability

The original contributions presented in this study are included in the article. Further inquiries can be directed to the corresponding author. Additional data supporting the findings of this study are available upon reasonable request from the corresponding author, access may be subject to restrictions due to confidentiality or ethical considerations within the project DIVAGRI.

## Abbreviations

The following abbreviations are used in this manuscript:

SLECI	Self-Regulating, Low Energy, Clay-Based Irrigation
SICE	Subsurface Irrigation with Ceramic Emitters
CP-SDIL	Ceramic-Patch-Type Subsurface Drip Irrigation Line
SDI	Sub-surface drip irrigation
IWP	Irrigation water productivity
DIVAGRI	101000348–DIVAGRI “Revenue diversification pathways in Africa through bio-based and circular agricultural innovations”
